# Acceptability of the use of health related quality of life measurements for decision-making in healthcare science in Vietnam: a qualitative study

**DOI:** 10.1136/bmjopen-2023-082405

**Published:** 2024-08-22

**Authors:** Vu Quynh Mai, Hoang Van Minh, Lars Lindholm, Sun Sun, Giang Bao Kim, Klas-Göran Sahlén

**Affiliations:** 1 Centre for Population Health Sciences, Hanoi University of Public Health, Ha Noi, Viet Nam; 2 Department of Epidemiology and Global Health, Umeå University, Umea, Sweden; 3 Hanoi University of Public Health, Hanoi, Viet Nam; 4 Department of Learning, Informatics Management and Ethics Karolinska Institute, Stockholm, Sweden; 5 Hanoi Medical University, Hanoi, Viet Nam; 6 Epidemiology and Global Health, Umea Universitet, Umea, Sweden

**Keywords:** health economics, health policy, quality of life

## Abstract

**Abstract:**

**Objective:**

This study was conducted with the objective of exploring the usage of health-related quality of life (HRQOL) outcomes and willingness of health technology assessment (HTA) and public health stakeholders to use the EQ-5D-5L instrument in healthcare decision-making processes in Vietnam.

**Method:**

In this qualitative study, 11 interviews were held with key stakeholders involved in healthcare decision-making for HTA between June 2021 and June 2022. The interviewees included members of the Vietnamese pharmacoeconomic council and public-health professionals from a diverse array of regions of Vietnam. The data collection involved obtaining verbal consent, warm-up discussions and interviews conducted via Zoom, with subsequent verification by interviewees. The analysis employed a theoretical thematic approach, adopting a deductive methodology to identify and analyse underlying ideas and meanings within the empirical data.

**Results:**

This study highlights the general importance and viability of HRQOL measures, and more particularly the EQ-5D-5L instrument, in healthcare decision-making in Vietnam. Challenges have been identified, including insufficient recognition, interpretation, standardisation and educational initiatives relating to HRQOL measurements. This study advocates for official training programmes on HRQOL measurements, guidelines for the application of the EQ-5D-5L and an open HRQOL database in Vietnam. Concerns regarding validity and outcome variation in HRQOL measurements underline the necessity for continuous psychometric properties assessments and regular updates to national HRQOL data in the Vietnamese context.

**Conclusion:**

HRQOL outcomes are important, and Vietnamese stakeholders express a readiness to employ the EQ-5D-5L in healthcare decision-making, especially HTA. Nevertheless, HRQOL measurements, including the EQ-5D-5L, are currently inadequately used in Vietnam, and further efforts are required to improve utilisation.

**STRENGTHS AND LIMITATIONS OF THIS STUDY**

- The theoretical thematic approach with a deductive methodology ensures thorough data analysis.
- The study includes diverse regional perspectives from key stakeholders but may overlook other critical voices in healthcare.
- Prolonged engagement, triangulation and peer-debriefing were performed to ensure the study’s trustworthiness.

## Introduction

 Achieving universal health coverage involves ensuring equitable access to healthcare services and healthcare financial security for all, and addressing three core dimensions: the extension of health-insurance coverage to all, financial protection for patients and provision of essential healthcare services.^1^ Health insurance is a key part of achieving universal health coverage, particularly in an era characterised by rapid development of healthcare interventions. While these advancements expand the array of options that can be included in health-insurance programmes, they also require careful consideration regarding effective budget allocation. In this context, health technology assessments (HTA) are vital, as they enable decision-makers to weigh both the costs and health benefits of various interventions. Given the constraints of having limited resources, decision-makers in Vietnam must use an evidence-based approach to determine which technologies should be provided under Vietnamese social health insurance (SHI). In 2018, the Ministry of Health in Vietnam issued a decree establishing a set of principles and criteria for developing the SHI drug list, with a specific requirement of evidence of an HTA to support the inclusion of any new drug.^2^ The aim is to ensure that universal health coverage through SHI in Vietnam is effectively pursued.

Economic evaluations are critical components of HTA, and involve cost–benefit, cost-effectiveness and cost–utility analyses (CUA). Of these, CUA is often recommended since decision-makers must consider a technology’s impact on both survival and quality of life. Quality-adjusted life-year (QALY) is a commonly employed metric when performing a CUA as it captures both reduced morbidity (quality gains) and reduced mortality (quantity gains).^2^ While health-related quality of life (HRQOL) is not typically employed as a final health outcome in economic evaluations, it is frequently utilised to operationalise QALYs. HRQOL is commonly derived from self-reported data, emphasising the patient’s experience of treatment effects that impact their health and quality of life.^3^ HRQOL measurements can broadly be categorised into two main types: condition-specific measures, which explore how specific diseases affect HRQOL, and generic measures, which cover a broader spectrum of HRQOL, potentially impacted by any disease.^2^ HRQOL outcomes are generally presented in two formats: profile based and preference based. Profile-based outcomes describe multiple attributes related to an individual’s HRQOL while preference-based outcomes quantify HRQOL using values, often ranging from 0 (representing death) to 1 (representing full health).^2^ In the realm of HTA the generic approach, which yields preference-based HRQOL outcomes, is common, particularly for calculating QALYs in CUAs.

As the Vietnamese healthcare system advances towards universal health coverage, it is crucial that it has the ability to meet community healthcare needs. The application of HRQOL measures is vital in this effort, serving diverse purposes in healthcare sciences. HRQOL assessments offer a key means of comparing intervention impacts on different aspects of QOL^3^ and are particularly valuable in situations where complete cure or guaranteed survival are challenging, such as chronic diseases or cancers; here, HRQOL provides a clearer indication of the impact of a healthcare intervention.^3^ In contrast to traditional medicine’s focus on professional assessment of symptom relief, HRQOL outcomes may reveal issues that are equally or more significant to patients.^3^ Therefore, HRQOL assessments have a role that extends beyond HTA, playing a crucial part in the efforts of various healthcare sciences to achieve the goal of universal health coverage.

The use of HRQOL measurements in the healthcare sciences in Vietnam has increased in research years. A search of the PubMed database was conducted using the search terms “health-related quality of life” and “Vietnam” and yielded a total of 62 publications that incorporated HRQOL outcomes. Notably, the earliest publication employing HRQOL measures was from the year 2000. However, a remarkable increase in the usage of HRQOL outcomes in research was noted, with 48 publications since 2018. Of these 61% relied on generic HRQOL measures; among those the EQ-5D instrument was the most frequently employed, featuring in 36 publications. Detailed references for the 62 publications can be found in online supplemental material 1.

The EQ-5D is one of the three most common profile-based HRQOL measures.^2^ It comprises five questions that are designed to describe HRQOL across five dimensions of health. Responses to the five questions are assessed at either three levels (EQ-5D-3L) or five levels (EQ-5D-5L) of severity. The instrument includes a Visual Analogue Scale—the EQ VAS—which ranges from 0 (indicating the worst health state) to 100 (the best health state). The EQ-5D incorporates preference-based values for each health profile generated. This includes a Vietnamese-language version of the questionnaire, the Visual Analogue Scale and a set of values for each of 3125 health profiles.^4^ It is noteworthy that the EQ-5D is a recommended instrument for deriving HRQOL weights in the calculation of QALYs in not only Vietnam but several other countries, including Australia, the UK and various European nations.^5^,^7^ In addition, the use of EQ-5D in healthcare sciences has been reported worldwide.^8^

In Vietnam, an EQ-5D-5L set was introduced in 2020; so far, the EQ-5D-5L is the only profile-based HRQOL measurement available in the country.^4^ However, efforts to establish its credibility as a decision-making support tool are necessary. Additionally, it is vital to explore the willingness of healthcare policy-makers and researchers to incorporate EQ-5D-5L into their practice, and at present no evidence of this exists. As such, this study was conducted with the objective of exploring the acceptability of HRQOL outcomes and willingness of decision-makers and public health professionals to use the EQ-5D-5L in healthcare-related decision-making processes, for example, to assess the new drug to be included in the SHI drug list, in Vietnam.

## Method

In this qualitative study, we conducted one pilot interview and ten interviews from the live study with relevant stakeholders between June 2021 and June 2022. Data from all 11 interviews were used in the main research analysis.

### Informants

We used a purposive sampling method. Our focus was to engage with stakeholders who play pivotal roles in healthcare decision-making within the context of Vietnamese HTA. Specifically, we targeted members of the pharmacoeconomic council responsible for evaluating HTA for the inclusion of new drugs. The council representatives selected were purposely chosen due to their expertise in various domains, including health economics, healthcare finance, healthcare policy and strategy, pharmacies, and health insurance.^9^ Given the widespread utilisation of HRQOL measurements in public-health research, we also purposively recruited public-health professionals from a wide array of regions of Vietnam. We provided potential participants with information about the study and obtained their verbal consent before data were collected.

### The interview guide

Despite growing evidence in support of the credibility of HRQOL as a metric, there is still a lack of understanding regarding its usefulness in decision-making. To establish the basis for our study, we first sought out a relevant theoretical framework for the development of the interview guide. Taking inspiration from the work of Sekhon *et al*, we embraced their proposed definition and theoretical framework for the acceptability of healthcare interventions.^10^ That study encompassed seven crucial aspects of ‘acceptability’: affective attitude, burden, ethical considerations, intervention coherence, opportunity cost, perceived effectiveness and self-confidence.^10^ In our study, we adopted Sekhon *et al*’s theoretical framework and developed an interview guide that aligns with these aspects (table 1).

**Table 1:** Interview guide

	Description
1	To what extent do you perceive HRQOL outcomes and the EQ-5D-5L instrument as being able to fulfil their intended role in healthcare decision-making processes?
2	How do you feel about HRQOL outcomes and the EQ-5D-5L themselves, as well as their application in healthcare decision-making processes?
3	What types and levels of burden, if any, do you associate with the use of HRQOL outcomes and the EQ-5D-5L in healthcare decision-making?
4	What types and magnitudes of costs do you perceive are linked to the use of HRQOL outcomes and the EQ-5D-5L in healthcare decision-making?
5	How confident are you in your ability to use HRQOL outcomes and the EQ-5D-5L, especially in healthcare decision-making processes? Please explain your confidence level.
6	What ethical considerations are connected to the application of HRQOL outcomes and the EQ-5D-5L in healthcare decision-making? According to your personal system of morality and norms, how appropriate is their use?
7	Could you elaborate on your understanding of HRQOL outcomes and the EQ-5D-5L themselves, and their application in healthcare decision-making processes?

### Data collection

All interviews were conducted by the main author (MQV). Interviews began with a warm-up discussion where participants shared their experiences of HRQOL measurements and the EQ-5D-5L. If a respondent did not have any experience with either HRQOL measurements or the EQ-5D-5L, the interview would not proceed. The interview then proceeded based on the guide. Each interview concluded with an open-ended discussion, allowing additional insights, comments and suggestions on the topic to be voiced. All interviews were conducted in Vietnamese language and each lasted between 50 and 70 min. Due to geographical barriers, all interviews were conducted online in Vietnamese using the Zoom platform. The audio of each meeting was recorded using Zoom’s recording function. To familiarising with the data, MQV conducted a review of all audio data, including the warm-up discussion, main part of the interview and closing remarks. MQV was the only team member with full access to the data. The audio recordings were anonymised and transferred to a public health student for transcription into Vietnamese. MQV conducted a final verification to ensure the accuracy and completeness of the transcriptions. The transcripts were then shared with the interview participants, providing them an opportunity to review and validate the content, if desired. To facilitate collaboration among authors, MQV translated half of the transcripts into English, including transcripts of the interviews with at least one representative from each stakeholder group.

### Analysis

The transcribed interviews underwent analysis using a theoretical thematic approach.^11 12^ The analytical process followed a deductive methodology. Initially, MQV thoroughly reviewed the transcripts, concentrating on underlying meanings and recurring patterns and recorded analytical observations. Subsequently, MQV conducted coding of the transcripts using NVivo V.12, generating preliminary themes and accompanying analytical notes. Finally, MQV systematically reviewed and organised these preliminary themes (subthemes) into overarching themes. Extensive discussions concerning the codes, subthemes and overarching themes were held with the other coauthors to ensure a comprehensive analysis. The analysis adopted a latent approach, aimed at identifying and analysing underlying ideas and meanings within the empirical data.^11^ This study was part of the lead author’s (MQV) doctoral project. Consequently, the preliminary results were reviewed by senior researchers from the university (outside the authors’ team) to improve the quality of the analysis.

### Patient and public involvement

None.

## Results

We invited 13 potential respondents, but 2 declined to participate due to their inexperience with both HRQOL measurements and the EQ-5D-5L. After discussing data saturation among the authors, we concluded that the dataset, which comprises 11 interviews with 6 females and 5 males, is sufficient. Among the respondents, two were from the south of Vietnam, one was from the central part of the country and the remaining eight were from the capital in the north of Vietnam. Two main themes have been developed during the analysis: (1) HRQOL measurements are crucial for making healthcare decisions and (2) HRQOL measurements still have limitations that hinder their wider use in Vietnam (table 2).

**Table 2:** Thematic map of the study

Codes	Subthemes	Themes
HRQOL outcomes are effective in healthcare sciences.	HRQOL measurements are effective in healthcare sciences.	HRQOL measurements are crucial for making healthcare decisions.
The EQ-5D-5L is an effective generic HRQOL measurement tool.		
The EQ-5D-5L is easy to use and practical.		
HRQOL outcomes and the EQ-5D-5L are useful in HTA.	HRQOL measurements support healthcare decision-making, particularly in HTA.	
HRQOL outcomes and the EQ-5D-5L are important for policy-making.		
The use of HRQOL outcomes is a convention for HTA.	HRQOL measurements are common in practice.	
The use of the EQ-5D-5L is globally accepted in HTA and healthcare sciences.		
There is strong confidence in the use of HRQOL outcomes and the EQ-5D-5L for HTA.		
The EQ-5D-5L is commonly used for HTA in Vietnam.		
‘HRQOL’ is an ambiguous term.	Lack of recognition and interpretation of HRQOL measurements.	HRQOL measurements in Vietnam still have limitations that hinder their wider use in Vietnam.
There is limited knowledge of both HRQOL outcomes and the EQ-5D-5L.		
The efficacy of the EQ-5D-5L in decision-making for specific conditions is unclear.		
There is no standardised approach to data collection for HRQOL measures.	Lack of standardisation in HRQOL measurement.	
There is a lack of knowledge regarding how to interpret EQ-5D-5L results.		
There are fluctuations in EQ-5D-5L results.		
There is the potential for bias in the use of HRQOL measures and the EQ-5D-5L in decision-making.		
More education on HRQOL measurement is needed.	Areas for improving HRQOL measurement in healthcare sciences.	
HRQOL measurements should be integrated into healthcare policy.		
HRQOL measurements should be integrated into healthcare information systems.		

HRQOLhealth-related quality of lifeHTAhealth technology assessment

### Theme 1: HRQOL measurements are crucial for making healthcare decisions

This theme highlights the crucial role of HRQOL measurements in healthcare sciences, particularly in the context of HTA. HRQOL measurements are a vital tool that greatly facilitates healthcare decision-making processes in Vietnam.

#### Subtheme 1.1: HRQOL measurements are effective in the healthcare sciences

HRQOL measurements are useful in evaluating the impact of healthcare interventions and play a pivotal role in elaborating clinical results and the effects of interventions. Additionally, HRQOL outcomes provide valuable insights from the perspectives of patients. While HRQOL outcomes are commonly employed in economic evaluations, their application could be extended to various study types, including intervention assessments and epidemiology studies.

The EQ-5D-5L is a common instrument for producing generic HRQOL outcomes among our informants. Despite its concise design, the EQ-5D-5L shows sensitivity in distinguishing HRQOL variations among subpopulations. However, some interviewees (in particular public-health researchers and pharmacists) expressed concern regarding the ability of the EQ-5D-5L to adequately capture the unique conditions of individuals; in spite of this, the consensus among the interviewees was that it enjoys widespread.

All participants agreed on the ease of use of the EQ-5D-5L. The instrument offers several notable advantages, including its brevity, which saves resources and ensures that surveys are conducted efficiently. The recent introduction of Vietnamese preference-based values for the EQ-5D-5L has strengthened its credibility, further facilitating its application in research conducted in Vietnam.

We used the EQ-5D-5L to assess the HRQOL of smokers in Hanoi and followed up with them over a period of two years. We observed changes in their HRQOL. Therefore, these results can be used to support policy-makers in prioritising policies that encourage smoking cessation. (Interviewed public-health researcher)

#### Subtheme 1.2: HRQOL measurements support healthcare decision-making, particularly with regard to HTA

Our informants told us that HRQOL outcomes are systematically integrated into HTA for well-informed decision-making and are used to explain the advantages of healthcare technologies to diverse audiences. These outcomes, which are essential for calculating QALYs, facilitate the estimation of long-term health benefits associated with healthcare technologies. In the Vietnamese context, the EQ-5D-5L is the only validated instrument for deriving HRQOL values for QALY estimation, underscoring its significance in HTA in Vietnam.

Within healthcare decision-making, HRQOL measurements, particularly in CUAs, have a pivotal role in determining the inclusion of drugs on the SHI reimbursement list in Vietnam. The informants suggested that the use of a generic HRQOL measure enables cross-disease comparison, aiding in resource-allocation decisions. Furthermore, HRQOL outcomes provide valuable insights into the impact of diseases on quality of life, facilitating the prioritisation of concerns within the healthcare system. In essence, HRQOL outcomes significantly contribute to shaping healthcare decision-making processes in Vietnam.

The EQ-5D is a suitable and effective tool for assessing an individual’s HRQOL. When necessary, it can be employed to estimate QALYs, and I personally prefer evidence presented as precise numerical data. I believe that utilising HRQOL outcomes in decision-making is entirely appropriate. (Interviewed healthcare strategist)

#### Subtheme 1.3: HRQOL measurements are common in practice

The interviewees felt that HRQOL outcomes for QALY estimation are globally accepted. QALYs are routinely employed in economic evaluations for HTA on an international scale. Consequently, the integration of HRQOL outcomes is widely embraced by HTA bodies worldwide, including those in Vietnam. The interviewees who are health economists and healthcare managers within HTA bodies stated that they have confidence in using HRQOL outcomes, particularly generic measures such as the EQ-5D-5L. This confidence stems from the ability of generic HRQOL outcomes to facilitate cross-context comparisons and the globally strong reputation of the EQ-5D-5L.

The EQ-5D, which assesses using either three or five levels of severity, is commonly recommended for HTA in a variety of countries due to its transparency and standardisation. The efficacy of the EQ-5D method of measuring health values is widely acknowledged. In Vietnam, policy-makers have expressed a strong interest in establishing HTA standardisation, emphasising the need for well-defined health outcomes with broad applicability to facilitate comparisons across interventions, fields and specific characteristics. The inclusion of the EQ-5D-5L in national HTA guidelines aligns well with these objectives. The interviewees acknowledged that the extensive use of the EQ-5D-5L in health sciences may serve as a robust reference source. Consequently, the EQ-5D-5L has recently been adopted in Vietnam as standard practice, contributing significantly to the establishment of HTA practices.

Acceptance of the EQ-5D-5L is widespread globally, paving the way for Vietnamese HTA stakeholders to embrace this measure. (Interviewed health-insurance expert)

### Theme 2: HRQOL measurements in Vietnam still have limitations that hinder their wider use here

This theme highlights the challenges associated with the recognition and standardisation of HRQOL measurements in Vietnam. It also indicates that there are opportunities to improve the application of HRQOL measurements in the country.

#### Subtheme 2.1: lack of recognition and interpretation of HRQOL measurements

The interviewees described a sense of ambiguity surrounding the theoretical framework for HRQOL due to the absence of a final definition or standardised measure of HRQOL. Each measurement instrument has its own definition and method for obtaining HRQOL, contributing to confusion among users. Despite the widespread international use of HRQOL measurements, doubts persist regarding their appropriateness among Vietnamese HTA stakeholders.

The concept of HRQOL is relatively new in Vietnam’s research landscape, and efforts to localise the EQ-5D-5L for Vietnam are still in early stages. Consequently, HRQOL measurements, including the EQ-5D-5L, are not well recognised in clinical and public health studies in Vietnam. The interviewed public-health researchers, unless strongly aligned with study objectives, show a disinclination to incorporate HRQOL outcomes in their studies. The interviewed public-health researchers and pharmacists expressed more interest in straightforward outcomes such as survival and clinical outcomes such as blood pressure.

The interviewees mentioned that the vague issue related to the HRQOL measurements and the lack of awareness on the application of HRQOL outcomes among their colleagues creates a significant challenge in explaining HRQOL and justifying the selection of the EQ-5D-5L among various HRQOL measurements. Moreover, the application of a generic HRQOL measurement such as the EQ-5D-5L to disease-specific studies may cause confusion, necessitating extensive efforts to explain it during its usage.

The concept of HRQOL is not well defined, making it challenging to convince my manager about its significance as a final health outcome. (Interviewed pharmacist)

#### Subtheme: lack of standardisation in HRQOL measurements

Standardisation in HRQOL measurements is crucial, yet clear instructions for Vietnamese researchers are lacking. Given that most HRQOL measurement tools rely on self-reported data, the standardisation of data collection is even more significant, encompassing considerations such as timing of data collection and management of data variations.

The absence of clear instructions on HRQOL outcomes in Vietnam has led to practices differing between studies. The interviewed pharmacists expressed low confidence in using HRQOL outcomes, citing fluctuations, especially with the EQ-5D-5L. The instrument incorporates two different methods of measuring HRQOL: the rating scale (EQ VAS) and the time trade-off multiple attributes system (EQ-5D-5L values).^13^ This leads to confusion as HRQOL outcomes are not consistent between the two methods.

The interviewed pharmacist emphasised that the variation of HRQOL outcomes may contribute to publication bias. Minor changes in HRQOL outcomes could significantly impact the results of economic evaluations. The lack of instructions for HRQOL outcomes may lead to more favourable responses because of such fluctuations which are being considered for economic evaluations.

Despite the availability of HRQOL measurement tools in Vietnam, confusion persists regarding the interpretation of HRQOL results. For example, while the EQ-5D-5L is widely used, many informants expressed a lack of awareness as to how to fully leverage the multiple-attribute health-descriptive system, stating that they instead tend to focus on EQ-5D-5L values. This may result from the absence of guidelines on interpreting HRQOL outcomes, including EQ-5D-5L outcomes, in Vietnam.

The EQ-5D-5L outcomes can vary due to differing collection times. For example, in the case of a follow-up survey, if data is collected after the respondent has just won the lottery, the results may show a significantly higher outcome compared to his/her previous responses. (Interviewed pharmacist)

#### Subtheme: areas for improving HRQOL measurement in healthcare sciences

The interviewees often stated that they had acquired their knowledge of HRQOL measurements through conferences or short training, necessitating self-learning from international sources in order to conduct proper analysis and report results. This poses a significant limitation for most Vietnamese researchers, highlighting the importance of education in HRQOL measurements, including the EQ-5D-5L. The interviewed health insurance expert suggested training medical and public-health students in HRQOL measures as for her it is required a relatively modest investment.

I don’t have any difficulty converting the five EQ-5D-5L questions into a utility score, but I’m unsure of what the resulting value signifies. Does it indicate a favourable or unfavourable result? I believe we need to provide more education on this matter. (Interviewed public-health researcher)

Furthermore, it is imperative to integrate HRQOL measurements into policy. Policy-makers, who are generally keen on standardising HTA, require HRQOL measurements in national HTA guidelines. The EQ-5D-5L is viewed as a tool for producing generic, preference-based HRQOL outcomes to calculate QALYs. However, according to our informants, the standardisation of HRQOL measurements for policy integration requires an increase in current levels of attention and investment.

The interviewees expressed a desire for a rich source of HRQOL data. The interviewees shared their experience with the financial burden they have had during their independent data collection of HRQOL outcomes for their economic evaluations. Hence, the establishment of an open-source Vietnamese HRQOL database is beneficial to all HTA stakeholders in Vietnam. The interviewees generally felt that integrating HRQOL measurements into the national health-information system is affordable. Consequently, discussions have mentioned expectations of interviewees for the development of a standardised HRQOL database.

It presents an opportunity to include EQ-5D-5L in the healthcare system and establish a robust database for research purposes. However, the cost of integrating and standardizing its use within the system remains to be estimated. (A healthcare system specialist)

## Discussion

This study aimed to assess the acceptability of HRQOL outcomes and the willingness of healthcare policy-makers and researchers to use the EQ-5D-5L in decision-making processes in Vietnam. Two main themes emerged: (1) HRQOL measurements are crucial for making healthcare decisions and (2) HRQOL measurements in Vietnam still have limitations that hinder their wider use in Vietnam.

The findings reveal that HRQOL measurements play a vital role in healthcare sciences, particularly in the context of HTA in Vietnam. These measurements effectively assess the impact of healthcare interventions, offering valuable insights from the perspectives of patients. This aligns with the findings of publications that have used HRQOL outcomes to assess the effectiveness of healthcare interventions in Vietnam.^14^,^17^ The EQ-5D-5L possesses widespread acceptance among our informants due to its brevity and sensitivity in distinguishing outcomes among subpopulations, making it a valuable tool for HTA in Vietnam. Our findings suggest that HRQOL outcomes play a supporting role in decision-making, especially in the HTA. They facilitate the calculation of QALYs in HTA, which in turn are used as evidence to support the inclusion of drugs on the SHI reimbursement list in Vietnam. The interviewees, particularly the health economists and healthcare managers, expressed strong confidence in using HRQOL outcomes, including EQ-5D-5L outcomes, for HTA due to the global prevalence of the EQ-5D-5L. Given its nature of generic attribute, EQ-5D is recommended for use in HTA in Australia, the UK and various European countries^5^,^7^ and the use of EQ-5D-5L has been recommended as it increases sensitivity and reduces ceiling effects in the final outcome.^18^

Challenges relating to the recognition and standardisation of HRQOL measurements still exist in Vietnam. Ambiguity surrounds the method of HRQOL measurements due to the absence of a definitive definition of HRQOL or standardised measurement method, causing confusion for the Vietnamese informants, which are aligned with the international literature.^19 20^ The interviewees indicated that the concept of HRQOL is relatively new in Vietnam, and HRQOL measurements, including the EQ-5D-5L, are, therefore, not well recognised in clinical and public-health studies. The findings presented in this study also suggest limited awareness of how to interpret HRQOL results, particularly those generated by the EQ-5D-5L, in Vietnam. Our informants believe that standardisation of HRQOL measurements is lacking in Vietnam, causing variations in data collection, analysis and result-interpretation practices. The interviewees also described an absence of clear instructions for using the HRQOL measures, which may introduce publication bias among studies that use HRQOL outcomes among Vietnamese researchers. Additionally, Vietnamese researchers have limited access to official education on HRQOL measurements and must rely on self-learning from international sources. Although it has been initiated, the integration of HRQOL measurements into policy requires an increase in current levels of attention and investment. Vietnamese stakeholders want to establish an open-source HRQOL database to ease the cost burden associated with independent data collection and improve the standardisation of HRQOL outcomes.

This study has several concrete recommendations. While we have confirmed the established role of HRQOL measurements in HTA, it has become evident that their application extends beyond the realm of HTA, which is in line with international literature.^21^ A review of studies that used HRQOL outcomes in Vietnam (online supplemental material 1) reveals that most were not specifically designed for health economic evaluations. This information can assist policy-makers in ensuring that the priorities of the Vietnamese healthcare system are aligned with the needs of the population. However, the review also highlighted that only a few research groups have consistently focused on HRQOL measures (online supplemental material 1). This suggests that there is a need to improve the recognition and interpretation of HRQOL measurements and provide education on the subject. The recommendation is to provide official training in medical and public-health programmes regarding HRQOL measurements, including both measurement methods and outcome classifications. Moreover, disseminating HRQOL-related knowledge through national conferences or publications can play a crucial role in raising public awareness. In addition, the study revealed that our informants face challenges in interpreting EQ-5D-5L results, indicating a knowledge gap. To address this, it is recommended that Vietnamese-language guidelines for the application of the EQ-5D-5L, covering aspects from design to reporting, be developed. This will serve as a valuable resource to bridge existing knowledge gaps and ensure a more effective application of the EQ-5D-5L in healthcare research and decision-making processes.

Despite a few published evidence regarding the credibility of HRQOL measurements in Vietnam,^422^,^25^ the matter of its psychometric properties is still discussed. Concerns persist regarding the validity, sensitivity and applicability of HRQOL measurements, including the EQ-5D-5L, across disease contexts. The appropriateness of a generic, preference-based HRQOL measurement such as the EQ-5D for specific condition contexts is widely debated, and some suggestions propose combining a generic measurement with condition-specific measurements for greater transparency.^26^ However, there is also the necessity for additional psychometric property assessments of HRQOL instruments. While evidence on the psychometric properties of the EQ-5D-5L, including validity, sensitivity and applicability, is abundant in the international literature,^27^,^33^ it remains limited in the context of Vietnam.^23 25^ In general, further efforts on these topics should be made in order to improve the utilisation of the EQ-5D-5L in Vietnam.

Concerns about the validity of the EQ-5D-5L, specifically differences between EQ VAS and EQ-5D-5L values, were raised by the interviewees. However, EQ VAS and the EQ-5D-5L are distinct methods with potentially different responses.^34^ In addition, the reliability of the EQ-5D-5L is questioned; the informants described the EQ-5D-5L producing different observations on the same objects. This is a significant factor in the hesitation expressed by the informants to use the EQ-5D-5L. However, this outcome variation can have several causes, such as non-standardised data collection (eg, interviewer bias, observation bias), unqualified samples (eg, small sample size, selection bias) or health fluctuations. Evidence supporting the reliability of the EQ-5D-5L has been published in several countries^35^,^37^ but not in Vietnam. It is important to note that outcome variation is sometimes expected in clinical studies (eg, trials) as a reflection of fluctuations in a patient’s health. In summary, efforts are required as regards internal validity and reliability testing for the EQ-5D-5L in Vietnam.

The need for a standardised approach to implementing HRQOL measurements was repeatedly mentioned by the informants; a potential solution to this is the establishment of a national HRQOL database. Therefore, it is recommended that HRQOL data for the Vietnamese population be added to a database that encompasses both general HRQOL outcomes and those of specific subpopulations. Additionally, standardised protocols for integrating HRQOL measurements into the national healthcare system would be beneficial for all stakeholders. The practice of using the EQ-5D as a routine, patient-reported outcome measure in healthcare has already been used in Canada and Sweden.^38 39^

This study addressed a topic that, to our knowledge, has not been explored in previous research. This unique quality may lead to questions about the trustworthiness of our study, which we would, therefore, like to discuss so as to ensure the confidence of readers in our findings. First, trust value is a critical aspect of research that pertains to a study’s ability to accurately capture the intended research subject.^40^ Essentially, trust value signifies the credibility of the study, relating to whether it has accurately and reliably represented the area of interest.^40^ Our study employed several strategies, including prolonged engagement, triangulation and peer-debriefing to this end. We spent extended periods in the field (prolonged engagement), conducting interviews, verifying information and engaging with interviewees to gather in-depth insights and feedback. In addition, we gathered data from various perspectives (triangulation), including health economists, pharmacists, public-health researchers, health-insurance managers and healthcare-strategy managers, all of whom contributed to our understanding of the use of HRQOL measures in decision-making in Vietnam. We followed the practice of peer-debriefing, which involves presenting preliminary results to experienced researchers for critical comments. This process allowed us to refine our findings and ensure their robustness. Additionally, we acknowledge that the transferability of our findings depends on the reader’s interpretation, as information applicability cannot solely rely on authors. We tried to provide a detailed description of the context in which our study was conducted.

This study has certain limitations that warrant consideration. First, with regard to the credibility of the truth value presented in this study, we did not conduct negative case analysis or member checks. Negative case analysis involves deliberately search for data that may contradict one’s working objectives within the existing dataset while member checks involve sharing results with study participants for their agreement or further discussion. However, there is no golden checklist for trustworthiness,^40^ and the absence of negative case analysis and member checks does not significantly affect the credibility of this study. Second, the coding process was performed solely by MQV, potentially impacting the confirmability of the data, as there was no audit conducted. We sought to address this limitation by engaging in extensive discussions regarding the codes and thematic system among the authors. Lastly, while we emphasised triangulation by obtaining data from diverse perspectives, there is a notable absence of input from hospital managers, clinicians and representatives from other Ministry of Health management units, such as information systems and data storage. This gap is acknowledged and represents a limitation in the broader context of the study.

### Conclusion

This study highlights the general importance and viability of HRQOL measures, and more particularly, the EQ-5D-5L instrument, in healthcare decision-making in Vietnam. Challenges have been identified, including insufficient recognition, standardisation and educational initiatives relating to HRQOL measurements. The study advocates for official training programmes on HRQOL measurements, guidelines for the application of the EQ-5D-5L and an open HRQOL database. Concerns regarding validity and outcome fluctuations in HRQOL measurements underline the necessity for continuous psychometric assessments and regular updates to national HRQOL data in the Vietnamese context.

## supplementary material

10.1136/bmjopen-2023-082405 online supplemental file 1

## Data Availability

Data are available on reasonable request.
