# Supplementary material for: Acceptability of the use of health related quality of life measurements for decision-making in healthcare science in Vietnam: a qualitative study
Source: BMJ Open. 2024 Aug 22;14(8):e082405. doi: 10.1136/bmjopen-2023-082405 (PMC11344514; doi:10.1136/bmjopen-2023-082405)
Supplement: online supplemental file 1 [file bmjopen-14-8-s001.pdf]

Supplement 1

62 publications that utilized HRQOL outcomes. Among these, one studies employed a single global measure (1), seven studies used condition-specific measures (2-8), 16 studies used generic profile-based measures (9-24), and 38 studies used generic preference-based measures (25-62). Notably, among studies employing generic preference-based HRQOL measurement, the EQ-5D—innovated by the EuroQol Research Foundation—stood out as the most frequently employed, with 17 publications discovered between 2010 and 2018 (25-41). Since the introduction of the Vietnamese preference-based EQ-5D-5L study on November 27, 2018, and its subsequent publication in 2020 (63), an additional 19 publications utilizing EQ-5D-5L have surfaced as of 2023 (42-60). Currently, EQ-5D-5L stands as the sole instrument capable of generating the HRQOL metric based on preferences of the general Vietnamese populace [14].

References

1. Vu DN, Phan DT, Nguyen HC, Le LTH, Nguyen HC, Ha TH, et al. Impacts of Digital Healthy Diet Literacy and Healthy Eating Behavior on Fear of COVID-19, Changes in Mental Health, and Health-Related Quality of Life among Front-Line Health Care Workers. Nutrients. 2021 Jul 30;13(8).
2. Tran BX. Quality of life outcomes of antiretroviral treatment for HIV/AIDS patients in Vietnam. PLoS One. 2012;7(7):e41062.
3. Tran BX, Nguyen LT, Do CD, Nguyen QL, Maher RM. Associations between alcohol use disorders and adherence to antiretroviral treatment and quality of life amongst people living with HIV/AIDS. BMC Public Health. 2014 Jan 10;14:27.
4. Nguyen HT, Collins PF, Pavey TG, Nguyen NV, Pham TD, Gallegos DL. Nutritional status, dietary intake, and health-related quality of life in outpatients with COPD. Int J Chron Obstruct Pulmon Dis. 2019;14:215-26.
5. Doan KVD, Nguyen HTM, Nguyen NTH, Dang KC, Yang SH, Duong TV. Associations of Socio-Demographic, Clinical and Biochemical Parameters with Healthcare Cost, Health- and Renal-Related Quality of Life in Hemodialysis Patients: A Clinical Observational Study. Int J Environ Res Public Health. 2020 Sep 9;17(18).
6. Phuong NTT, Ngoc VTN, Linh LM, Duc NM, Tra NT, Anh LQ. Bruxism, Related Factors and Oral Health-Related Quality of Life Among Vietnamese Medical Students. Int J Environ Res Public Health. 2020 Oct 12;17(20).
7. Nguyen HP, Pham DD, Dinh Nguyen D, Nguyen PV, Bui VA, Hoang MT, et al. Evaluating the Safety and Quality of Life of Colorectal Cancer Patients Treated by Autologous Immune Enhancement Therapy (AIET) in Vinmec International Hospitals. Int J Mol Sci. 2022 Sep 26;23(19).
8. Nguyen HTH, Duong KL, Nguyen ST, Trinh Q, Hoang HTL, Phung TQ, et al. Quality of Life and Its Associated Factors Among Cancer Patients Receiving Chemotherapy at Oncology Hospitals in Vietnam After the Third Wave of the COVID-19 Pandemic. Cancer Manag Res. 2022;14:2429-44.
9. Le MT, Holton S, Nguyen HT, Wolfe R, Fisher J. Victimisation, poly-victimisation and health-related quality of life among high school students in Vietnam: a cross-sectional survey. Health Qual Life Outcomes. 2016 Nov 4;14(1):155.
10. Dao-Tran TH, Seib C, Jones L, Anderson D. A cross-cultural comparison of health-related quality of life and its associated factors among older women in Vietnam and Australia. BMC Res Notes. 2018 Mar 13;11(1):174.
11. Tran TH, Trinh NL, Hoang Y, Nguyen TL, Vu TT. Health-Related Quality of Life Among Vietnamese Breast Cancer Women. Cancer Control. 2019 Jan-Dec;26(1):1073274819862787.

12. Nguyen HC, Nguyen MH, Do BN, Tran CQ, Nguyen TTP, Pham KM, et al. People with Suspected COVID-19 Symptoms Were More Likely Depressed and Had Lower Health-Related Quality of Life: The Potential Benefit of Health Literacy. J Clin Med. 2020 Mar 31;9(4).
13. Pham TB, Nguyen TT, Truong HT, Trinh CH, Du HNT, Ngo TT, et al. Effects of Diabetic Complications on Health-Related Quality of Life Impairment in Vietnamese Patients with Type 2 Diabetes. J Diabetes Res. 2020;2020:4360804.
14. Stevens SM, Gustavson DE, Fang B, Tu X, Logue M, Lyons MJ, et al. Predicting Health-Related Quality of Life in Trauma-Exposed Male Veterans in Late Midlife: A 20 Year Longitudinal Study. Int J Environ Res Public Health. 2020 Jun 24;17(12).
15. Tran BX, Moir M, Nguyen TMT, Do HN, Vu GT, Dang AK, et al. Changes in quality of life and its associated factors among illicit drug users in Vietnamese mountainous provinces: a 12-month follow-up study. Subst Abuse Treat Prev Policy. 2020 Mar 18;15(1):23.
16. Tran TV, Nguyen HC, Pham LV, Nguyen MH, Nguyen HC, Ha TH, et al. Impacts and interactions of COVID-19 response involvement, health-related behaviours, health literacy on anxiety, depression and health-related quality of life among healthcare workers: a cross-sectional study. BMJ Open. 2020 Dec 7;10(12):e041394.
17. Nguyen DT, Dang TC, Nguyen QA, Le TD, Hoang TD, Tran TNT, et al. The effect of subcutaneous injection of methylprednisolone acetate and lidocaine for refractory postherpetic neuralgia: a prospective, observational study. Health Sci Rep. 2021 Jun;4(2):e271.
18. Nguyen MH, Pham TTM, Nguyen KT, Nguyen YH, Tran TV, Do BN, et al. Negative Impact of Fear of COVID-19 on Health-Related Quality of Life Was Modified by Health Literacy, eHealth Literacy, and Digital Healthy Diet Literacy: A Multi-Hospital Survey. Int J Environ Res Public Health. 2021 May 6;18(9).
19. Tran Kien N, Phuong Hoa N, Minh Duc D, Wens J. Health-related quality of life and associated factors among patients with type II diabetes mellitus: A study in the family medicine center (FMC) of Agricultural General Hospital in Hanoi, Vietnam. Health Psychol Open. 2021 Jan-Jun;8(1):2055102921996172.
20. Vu LN, Nghia NQ, Tuan TM, Phuong TH, Vo HL, Viet KN, et al. Measuring Health-Related Quality of Life in Vietnamese Patients After Kidney Transplantation. Front Surg. 2021;8:646629.
21. Yoon S, An S, Noh DH, Tuan LT, Lee J. Effects of health education on adolescents' non-cognitive skills, life satisfaction and aspirations, and health-related quality of life: A cluster-randomized controlled trial in Vietnam. PLoS One. 2021;16(12):e0259000.
22. Pham TTM, Vu MT, Luong TC, Pham KM, Nguyen LTK, Nguyen MH, et al. Negative Impact of Comorbidity on Health-Related Quality of Life Among Patients With Stroke as Modified by Good Diet Quality. Front Med (Lausanne). 2022;9:836027.
23. Phan HT, Reeves MJ, Gall S, Morgenstern LB, Xu Y, Lisabeth LD. Factors Contributing to Sex Differences in Health-Related Quality of Life After Ischemic Stroke: BASIC (Brain Attack Surveillance in Corpus Christi) Project. J Am Heart Assoc. 2022 Sep 6;11(17):e026123.
24. Ngo-Metzger Q, Sorkin DH, Mangione CM, Gandek B, Hays RD. Evaluating the SF-36 Health Survey (Version 2) in Older Vietnamese Americans. J Aging Health. 2008 Jun;20(4):420-36.
25. Hoi le V, Chuc NT, Lindholm L. Health-related quality of life, and its determinants, among older people in rural Vietnam. BMC Public Health. 2010 Sep 11;10:549.
26. Tran BX, Ohinmaa A, Nguyen LT. Quality of life profile and psychometric properties of the EQ-5D-5L in HIV/AIDS patients. Health Qual Life Outcomes. 2012 Nov 1;10:132.
27. Tran BX, Nguyen LH, Phan HT, Nguyen LK, Latkin CA. Preference of methadone maintenance patients for the integrative and decentralized service delivery models in Vietnam. Harm Reduct J. 2015 Sep 17;12:29.

28. Tran BX, Nguyen LH, Nguyen CT, Phan HT, Latkin CA. Alcohol abuse increases the risk of HIV infection and diminishes health status of clients attending HIV testing services in Vietnam. Harm Reduct J. 2016 Feb 16;13:6.
29. Tran BX, Nguyen LH, Nong VM, Nguyen CT. Health status and health service utilization in remote and mountainous areas in Vietnam. Health Qual Life Outcomes. 2016 Jun 7;14:85.
30. Tran BX, Nguyen LH, Nong VM, Nguyen CT, Phan HT, Latkin CA. Behavioral and quality-of-life outcomes in different service models for methadone maintenance treatment in Vietnam. Harm Reduct J. 2016 Feb 2;13:4.
31. Bang KS, Tak SH, Oh J, Yi J, Yu SY, Trung TQ. Health Status and the Demand for Healthcare among the Elderly in the Rural Quoc-Oai District of Hanoi in Vietnam. Biomed Res Int. 2017;2017:4830968.
32. Nguyen LH, Nguyen LHT, Boggiano VL, Hoang CD, Van Nguyen H, Le HT, et al. Quality of life and healthcare service utilization among methadone maintenance patients in a mountainous area of Northern Vietnam. Health Qual Life Outcomes. 2017 Apr 20;15(1):77.
33. Nguyen LH, Tran BX, Hoang Le QN, Tran TT, Latkin CA. Quality of life profile of general Vietnamese population using EQ-5D-5L. Health Qual Life Outcomes. 2017 Oct 11;15(1):199.
34. Tran BX, Huong LT, Hinh ND, Nguyen LH, Le BN, Nong VM, et al. A study on the influence of internet addiction and online interpersonal influences on health-related quality of life in young Vietnamese. BMC Public Health. 2017 Jan 31;17(1):138.
35. Dang AK, Nguyen LH, Nguyen AQ, Tran BX, Tran TT, Latkin CA, et al. Physical activity among HIV-positive patients receiving antiretroviral therapy in Hanoi and Nam Dinh, Vietnam: a cross-sectional study. BMJ Open. 2018 May 10;8(5):e020688.
36. Nguyen HTT, Moir MP, Nguyen TX, Vu AP, Luong LH, Nguyen TN, et al. Health-related quality of life in elderly diabetic outpatients in Vietnam. Patient Prefer Adherence. 2018;12:1347-54.
37. Nguyen LT, Alexander K, Yates P. Psychoeducational Intervention for Symptom Management of Fatigue, Pain, and Sleep Disturbance Cluster Among Cancer Patients: A Pilot Quasi-Experimental Study. J Pain Symptom Manage. 2018 Jun;55(6):1459-72.
38. Tran BX, Dang AK, Truong NT, Ha GH, Nguyen HLT, Do HN, et al. Depression and Quality of Life among Patients Living with HIV/AIDS in the Era of Universal Treatment Access in Vietnam. Int J Environ Res Public Health. 2018 Dec 17;15(12).
39. Tran BX, Moir MP, Thai TPT, Nguyen LH, Ha GH, Nguyen THT, et al. Socioeconomic Inequalities in Health-Related Quality of Life among Patients with Cardiovascular Diseases in Vietnam. Biomed Res Int. 2018;2018:2643814.
40. Tran BX, Nguyen HLT, Le QNH, Mai HT, Ngo C, Hoang CD, et al. Alcohol and tobacco use among methadone maintenance patients in Vietnamese rural mountainside areas. Addict Behav Rep. 2018 Jun;7:19-25.
41. Tran BX, Thu Vu G, Hoang Nguyen L, Tuan Le Nguyen A, Thanh Tran T, Thanh Nguyen B, et al. Cost-of-Illness and the Health-Related Quality of Life of Patients in the Dengue Fever Outbreak in Hanoi in 2017. Int J Environ Res Public Health. 2018 Jun 5;15(6).
42. Khue PM, Thom VT, Minh DQ, Quang LM, Hoa NL. Depression and Anxiety as Key Factors Associated With Quality of Life Among Lung Cancer Patients in Hai Phong, Vietnam. Front Psychiatry. 2019;10:352.
43. Ngo CQ, Phan PT, Vu GV, Pham QLT, Nguyen LH, Vu GT, et al. Effects of Different Comorbidities on Health-Related Quality of Life among Respiratory Patients in Vietnam. J Clin Med. 2019 Feb 7;8(2).
44. Nguyen AT, Nguyen LH, Nguyen TX, Nguyen HTT, Nguyen TN, Pham HQ, et al. Frailty Prevalence and Association with Health-Related Quality of Life Impairment among Rural Community-Dwelling Older Adults in Vietnam. Int J Environ Res Public Health. 2019 Oct 12;16(20).

45. Nguyen SH, Nguyen LH, Vu GT, Nguyen CT, Le THT, Tran BX, et al. Health-Related Quality of Life Impairment among Patients with Different Skin Diseases in Vietnam: A Cross-Sectional Study. Int J Environ Res Public Health. 2019 Jan 23;16(3).
46. Nguyen TS, Nguyen TLH, Pham TTV, Hua S, Ngo QC, Li SC. Impact of pharmaceutical care in the improvement of medication adherence and quality of life for COPD patients in Vietnam. Respir Med. 2019 Jul;153:31-7.
47. Pham KTH, Nguyen LH, Vuong QH, Ho MT, Vuong TT, Nguyen HT, et al. Health Inequality between Migrant and Non-Migrant Workers in an Industrial Zone of Vietnam. Int J Environ Res Public Health. 2019 Apr 28;16(9).
48. Vu HM, Dang AK, Tran TT, Vu GT, Truong NT, Nguyen CT, et al. Health-Related Quality of Life Profiles among Patients with Different Road Traffic Injuries in an Urban Setting of Vietnam. Int J Environ Res Public Health. 2019 Apr 24;16(8).
49. Vu HM, Nguyen LH, Tran TH, Pham KTH, Phan HT, Nguyen HN, et al. Effects of Chronic Comorbidities on the Health-Related Quality of Life among Older Patients after Falls in Vietnamese Hospitals. Int J Environ Res Public Health. 2019 Sep 27;16(19).
50. Vu TTM, Le TV, Dang AK, Nguyen LH, Nguyen BC, Tran BX, et al. Socioeconomic Vulnerability to Depressive Symptoms in Patients with Chronic Hepatitis B. Int J Environ Res Public Health. 2019 Jan 17;16(2).
51. Manh Than H, Minh Nong V, Trung Nguyen C, Phu Dong K, Ngo HT, Thu Doan T, et al. Mental Health and Health-Related Quality-of-Life Outcomes Among Frontline Health Workers During the Peak of COVID-19 Outbreak in Vietnam: A Cross-Sectional Study. Risk Manag Healthc Policy. 2020;13:2927-36.
52. Nguyen LH, Thu Vu G, Ha GH, Tat Nguyen C, Vu HM, Nguyen TQ, et al. Fear of Falling among Older Patients Admitted to Hospital after Falls in Vietnam: Prevalence, Associated Factors and Correlation with Impaired Health-Related Quality of Life. Int J Environ Res Public Health. 2020 Apr 6;17(7).
53. Tran BT, Pham NH, Nguyen TX, Choi KS, Sohn DK, Kim SY, et al. Measurement of Health-Related Quality of Life Among Colorectal Cancer Patients Using the Vietnamese Value Set of the EQ-5D-5L. Patient Prefer Adherence. 2020;14:2427-37.
54. Tran BX, Nguyen HT, Le HT, Latkin CA, Pham HQ, Vu LG, et al. Impact of COVID-19 on Economic Well-Being and Quality of Life of the Vietnamese During the National Social Distancing. Front Psychol. 2020;11:565153.
55. Vu MQ, Tran TTP, Hoang TA, Khuong LQ, Hoang MV. Health-related quality of life of the Vietnamese during the COVID-19 pandemic. PLoS One. 2020;15(12):e0244170.
56. Ngo AT, Nguyen LH, Dang AK, Hoang MT, Nguyen THT, Vu GT, et al. Bullying experience in urban adolescents: Prevalence and correlations with health-related quality of life and psychological issues. PLoS One. 2021;16(6):e0252459.
57. Tien NLB, Thanh VV, Hanh KTH, Anh PG, Huyen LTM, Tu NT, et al. Quality of Life and Activities of Daily Living among Patients with Complete Cervical Spinal Cord Injury and Surgical Treatment in Vietnam. Int J Environ Res Public Health. 2021 Sep 15;18(18).
58. Huynh G, Nguyen BT, Nguyen HTN, Le NT, An PL, Tran TD. Health-Related Quality of Life Among Patients Recovered From COVID-19. Inquiry. 2022 Jan-Dec;59:469580221143630.
59. Ngan TT, Mai VQ, Van Minh H, Donnelly M, O'Neill C. Health-related quality of life among breast cancer patients compared to cancer survivors and age-matched women in the general population in Vietnam. Qual Life Res. 2022 Mar;31(3):777-87.
60. Vu H, Norman R, Pham NM, Nguyen HTT, Pham HM, Nguyen QN, et al. Sex differences in quality of life of patients following percutaneous coronary intervention in Vietnam. Qual Life Res. 2023 Jan;32(1):71-9.

61. Ha NT, Trang DTH, Ha LTT. Is obesity associated with decreased health-related quality of life in school-age children?-Results from a survey in Vietnam. AIMS Public Health. 2018;5(4):338-51.
62. Ngoc Thi Dang D, Ngoc Thi Nguyen L, Thi Dang N, Quang Dang H, Ta TV. Quality of Life in Vietnamese Gastric Cancer Patients. Biomed Res Int. 2019;2019:7167065.
63. Mai VQ, Sun S, Minh HV, Luo N, Giang KB, Lindholm L, et al. An EQ-5D-5L Value Set for Vietnam. Qual Life Res. 2020 Jul;29(7):1923-33.
