# Supplementary material for: Acceptability of the use of health related quality of life measurements for decision-making in healthcare science in Vietnam: a qualitative study
Source: BMJ Open. 2024 Aug 22;14(8):e082405. doi: 10.1136/bmjopen-2023-082405 (PMC11344514; doi:10.1136/bmjopen-2023-082405)
Supplement: Supplementary file 2 [file bmjopen-2023-082405.reviewer_comments.pdf]

## PEER REVIEW HISTORY

BMJ Open publishes all reviews undertaken for accepted manuscripts. Reviewers are asked to complete a checklist review form (**http://bmjopen.bmj.com/site/about/resources/checklist.pdf**) and are provided with free text boxes to elaborate on their assessment. These free text comments are reproduced below.

## ARTICLE DETAILS

| | |
|---|---|
| **TITLE (PROVISIONAL)** | Acceptability of the use of health-related quality of life measurements for decision-making in healthcare science in Vietnam: a qualitative study |
| **AUTHORS** | Vu, Mai; Van Minh, Hoang; Lindholm, Lars; Sun, Sun; Kim, Giang; Sahlén, Klas-Göran |

## VERSION 1 – REVIEW

| | |
|---|---|
| **REVIEWER** | Mazur, Ana<br>Bielefeld University, School of Public Health |
| **REVIEW RETURNED** | 04-Jan-2024 |

| | |
|---|---|
| **GENERAL COMMENTS** | Dear Authors,<br>Thank you for your paper and such an important research issue for your country. I made my review in form of comments in your PDF file. You find this attached.<br>Best regards,<br>Dr. Ana Mazur |

## VERSION 1 – AUTHOR RESPONSE

We would like to thank the reviewers for their time and constructive feedback. We have carefully reviewed your comments and made every effort to improve the text accordingly.
Reviewer 1
Page 6 of 25

1. "Line 7-16: It is neither stated who conducted the search nor how it resulted in 62 publications. Was there a search string? Were there search and selection kriteria? Was the purpose of this search the preparation of the actual study? It sounds like it belongs to methods. Please clarify this topic and provide this information at least in supplementary material 1."

*Response:*
We thank the reviewer for the comment. This search was conducted as part of the literature review for this study in early 2023. The purpose of the search was to identify available measurements for health-related quality of life (HRQOL) in Vietnam. This was intended to provide background information on the topic and was not part of the methodology for the study presented in this paper. Search string is simple as presented "health-related quality of life" and "Vietnam".

2. "Line 28-31: Please make a short summary of the evidence concerning the usage of EQ-5D in economic evaluation of interventions and why it is recommended in other countries. "

*Response:*

We thank the reviewer for the comment. We agree that a more detailed rationale for the application of the EQ-5D should be provided. However, due to word limitations, we have kept this information concise. We discussed the application of profile-based HRQOL in economic evaluations on lines 37-43 of page 5, and the use of the EQ-5D, one of the three most common profile-based HRQOL measurements worldwide, on lines 19-31 of page 6. We also addressed the popularity of the EQ-5D in real-world settings. We have clarified the text as follows:
*"The EQ-5D is one of the three most common profile-based HRQOL measures (2)."*
*"In Vietnam, an EQ-5D-5L set was introduced in 2020; so far, the EQ-5D-5L is the only profile-based HRQOL measurement available in the country (4)."*

3. "Line 34: Other preference measures are also used like HUI, QWB-SA. Why was EQ-5D with its European standards introduced in Vietnam? Could you please give us some more background on this point?"

<u>*Response:*</u>
We thank the reviewer for the comment. We agree that there are other HRQOL measurements that would be helpful. We introduced the EQ-5D rather than other instruments within the scope of this study, as the EQ-5D is the only index-based HRQOL measurement available in Vietnam. We have clarified this point in the text:
*"In Vietnam, an EQ-5D-5L set was introduced in 2020; so far, the EQ-5D-5L is the only profile-based HRQOL measurement available in the country (4)."*
Regarding your point on why the EQ-5D, based on European standards, was introduced in Vietnam, we would like to explain that the EQ-5D is designed to match universal standards for measuring health-related quality of life. The EQ-5D has been used worldwide, including in ASEAN countries such as Singapore, Thailand, and Indonesia. Therefore, the introduction of the EQ-5D in Vietnam follows common practice for this tool globally. We have clarified this point in the text as well:
*"In addition, the use of EQ-5D in healthcare sciences has been reported worldwide (8)."*

Page 7 of 25

4. "Line 4-5: Please provide the method of approach. " Please state if the interview guide was pilot tested. If so, describe any modifications.

<u>*Response:*</u>
We thank the reviewer for the comment. The sampling was done purposively, we added this information into the informant part.
Although a pilot study is not necessary for qualitative research, we conducted one pilot interview. The data from this pilot interview was subsequently included in the main research analysis. We have clarified the pilot process in the text as follows:
*"In this qualitative study, we conducted one pilot interview and ten interviews from the live study with relevant stakeholders between June 2021 and June 2022. Data from all eleven interviews were used in the main research analysis. "*

5. "Line 31-32: The purpose of this procedure is not clear enough. "

<u>*Response:*</u>
We believe that the reviewer mentioned to this section: "*All interviews were conducted in Vietnamese language using the Zoom platform and were audio-recorded. The interviews lasted between 50 and 70 minutes. MVQ conducted a review of all audio data, including the warm-up discussion, main part of the interview, and closing remarks."*
The purpose of this step is to help the coder (MVQ) familiarising with the data, she first conducted the interview, second re-listen the tape, and then read and re-read the transcription several times before coding. This process is part of the thematic analysis approach suggested by Braun and Clarke as referenced in source 11 in the text now (Braun V, Clarke V. Using thematic analysis in psychology. Qualitative Research in Psychology. 2006;3(2):77-101.), please refer to this table in Braun's paper.

**Table 1** Phases of thematic analysis

| Phase | Description of the process |
|---|---|
| 1. Familiarizing yourself with your data: | Transcribing data (if necessary), reading and re-reading the data, noting down initial ideas. |
| 2. Generating initial codes: | Coding interesting features of the data in a systematic fashion across the entire data set, collating data relevant to each code. |
| 3. Searching for themes: | Collating codes into potential themes, gathering all data relevant to each potential theme. |
| 4. Reviewing themes: | Checking if the themes work in relation to the coded extracts (Level 1) and the entire data set (Level 2), generating a thematic 'map' of the analysis. |
| 5. Defining and naming themes: | Ongoing analysis to refine the specifics of each theme, and the overall story the analysis tells, generating clear definitions and names for each theme. |
| 6. Producing the report: | The final opportunity for analysis. Selection of vivid, compelling extract examples, final analysis of selected extracts, relating back of the analysis to the research question and literature, producing a scholarly report of the analysis. |

6. "Line 31: Poor description of data management. Please make clear how audio was recorded, how data was stored, who in the whole team had access to audio and text material."

<u>*Response:*</u>

We thank the reviewer; we have clarified the data management in the text as follows:

"Due to geographical barriers, all interviews were conducted online in Vietnamese using the Zoom platform. The audio of each meeting was recorded using Zoom's recording function. To familiarising with the data, MVQ conducted a review of all audio data, including the warm-up discussion, main part of the interview, and closing remarks. MVQ was the only team member with full access to the data. The audio recordings were anonymized and transferred to a public health student for transcription into Vietnamese. MVQ conducted a final verification to ensure the accuracy and completeness of the transcriptions."

7. "Line 38-39: It is not clear what parts of the interviews were translated and why others not."

<u>*Response:*</u>

We thank the reviewer; we have clarified the data management in the text as follows:

*"To facilitate collaboration among authors, MVQ translated half of the transcripts into English, including transcripts of the interviews with at least one representative from each stakeholder group."*

8. "Line 43-54: The reference given (Dao-Tran et al., 2018) does not fit to any theoretical approach that coud have been used as basis for a deductive analysis.

<u>*Response:*</u>

We thank the reviewer for the comment. We believe the reviewer checked the list of references for Supplementary File 1 instead of the main list of references. The reference in line 43 of our text (now is #11) refers to Braun's approach to conducting thematic analysis (2006) also, we have added an updated knowledge related to thematic analysis in ref 12 (Braun's paper 2019).

Moreover, your description of conducted analysis corresponds to an inductive not a deductive process, e.g. ""generating preliminary themes"", ""organised sub-themes into overarching themes"", ""analysis adopted a latent approach, aimed at identifying and analysing underlying ideas and meanings"". Please clarify these essential points!"

<u>*Response:*</u>

We thank the reviewer for the comment. We believe that the steps mentioned by the reviewer pertain to the approach of conducting thematic analysis. Considering that we were inspired by Sekhon's theoretical framework to design the questions and that the themes system aligns well with Sekhon's framework, we consider a deductive process to be more suitable for our work.

9. "Lines 13-16: Please state why this study see EQ-5D as a healthcare intervention according to Sekhon's theoretical framework."

<u>*Response:*</u>

We thank the reviewer for the comment. We did not consider EQ-5D as a healthcare intervention. As discussed in the method section, there is no conceptual framework to assess the acceptability of a health outcome measurement. Therefore, we were inspired by Sekhon's theoretical framework for the components that should be considered when discussing "acceptability."

Page 8 of 25

10. "Please provide information about characteristic of interviewed experts, such as current position, area of expertise, years of work in the area, gender, age, etc.

<u>*Response:*</u>

We thank the reviewer for the comment. As half of our participants were members of, or involved with, the pharmacoeconomic council (our targeted group), including detailed information such as their current position, area of expertise, years of work in the field, gender, and age could reveal their identities, which we aim to avoid in this study. However, we have added a short description of our respondents in the results section as follows:

*"Overall, we successfully conducted eleven interviews with six females and five males. Among the respondents, two were from the south of Vietnam, one was from the central part of the country, and the remaining eight were from the capital in the north of Vietnam".*

11. The interview guide was based on a framework of acceptability of healthcare interventions. It is essential to report what kind of experience with HRQoL-outcomes and EQ-5D-5L these experts have. In other words, how far have been the interviewed experts ""exposed"" to these ""interventions"" they are supposed to assess? Recruting results are not clear enough, for example, state how many people were invited, how many refused to participate or dropped out."

<u>*Response:*</u>

We thank the reviewer for the comment. We clarified the information in the data collection part as follows:

"*Interviews began with a warm-up discussion where participants shared their experiences of HRQOL measurements and the EQ-5D-5L. If a respondent did not have any experience with either HRQOL measurements or the EQ-5D-5L, the interview would not proceed."*

And in the result part as follows:

"*We invited 13 potential respondents, but two declined to participate due to their inexperience with both HRQOL measurements and the EQ-5D-5L. Overall, we successfully conducted eleven interviews with six females and five males".*

12. "Lines 3-5: Again these lines show the analysis was conducted on an inductive basis."

<u>*Response:*</u>

We thank the reviewer for the comment. We followed a deductive thematic analysis approach.

Page 12 of 25

13. "Line 27-29: Please improve your discussion by reflecting if the reasons of recommendation of the instrument and concerns about it in other countries are similar or not. What makes EQ-5D more applicable than other methods?"

<u>*Response:*</u>

We thank the reviewer for the comment. In fact the reasons of recommendation of the instrument and concerns about it in other countries have not been well reported. However, we managed to add-on some information in the text as follows:

*"Given its nature of generic attribute, EQ-5D is recommended for use in HTA in Australia, the United Kingdom, and various European countries (5-7) and the use of EQ-5D-5L has been recommended as it increases sensitivity and reduces ceiling effects in the final outcome (17)."*

14. "Line 34-36: Which issues? Please draw parallel lines where possible.

<u>*Response:*</u>

We thank the reviewer for the comment. We revised the text as follows:

*“ Ambiguity surrounds the method of HRQOL measurements due to the absence of a definitive definition of HRQOL or standardised measurement method, causing confusion for the Vietnamese informants, which are align with the international literature (19, 20).”*

15. Moreover, your text suggests there are no critical voices (in science) concerning the use of HRQoL-intruments, like EQ-5D, in other countries. Consider reflecting the limitations of the instrument when it comes to assessing differences in quality of life among people with different social economic status or many other social inequalities. "

*Response:*

We thank the reviewer for the comment. In fact, there are several concerns related to HRQOL instruments in general, and EQ-5D specifically, in other countries, as cited in references 19 and 20. The limitations of these instruments in assessing quality of life by different socioeconomic statuses have been discussed elsewhere. However, this was not a concern among respondents in our study, so we did not address it within the scope of this study.

Page 13 of 25

16. "Line 56: Please provide examples that underpin your statement."

*Response:*

We thank the reviewer for the comment. We assumed that the reviewers would like to see more quotes regarding to our statement in line 56: “*The need for a standardised approach to implementing HRQOL measurements was repeatedly mentioned by the informants; a potential solution to this is the establishing of a national HRQOL database”.* However, it is not common to add quotes in the discussion section, so I would add a quote in the result part as follows:

*“It presents an opportunity to include EQ-5D-5L in the healthcare system and establish a robust database for research purposes. However, the cost of integrating and standardizing its use within the system remains to be estimated”.*

*-(A healthcare system specialist)*

Page 14 of 25

17. "Line 22: The perspective of several stakeholders on the topic can be a strength of the study. However, data triangulation would require more data diversity than interviews as only source. Moreover, triangulation was not described under methods. If this procedure has been carried out, please present it properly. Results would include which positions were taken by the different experts. A table could be helpful to visualise differences and similarities."

*Response:*

We thank the reviewer for the comment and agree that triangulation extends beyond just data sources. Data triangulation was not planned from the outset. We followed suggestions from Lars Dahlgren et al. in the book Qualitative Methodology for International Public Health (pages 42-49) to assess the trustworthiness of our analysis (refer to ref #40 now). Triangulation, as explained in the text, can occur through various means such as data sources, data collection methods, investigators, or research methodologies. In our reflection on the trustworthiness of our study, we emphasize that we incorporated data source triangulation to enhance the reliability of our findings.

18. "Lines 25-27: This procedure has not been presented under methods. Please present results related to the feedback of the experts to the preliminary results."

*Response:*

We thank the reviewer for the comment. We added information to the method as follows:

“*This study was part of the lead author's (MQV) doctoral project. Consequently, the preliminary results were reviewed by senior researchers from the university* (outside the authors' team) *to improve the quality of the analysis.”*

19. "Line 26-28: Which of the results have required redefinition according to the experts? Please provide more transparency."

*Response:*

We thank the reviewer for the comment. The expert provided critical feedback on how we shaped the overarching themes, prompting us to re-analyze the theme system. The present results are the final endpoints after several discussions among the authors' team and feedback from the university's experts. Due to the word limit for this article, we have summarized this process as in lines 26-28 of the text.

20. "Line 35-40: Please check item 32 under: https://cdn.elsevier.com/promis_misc/ISSM_COREQ_Checklist.pdf Please refer to a methodological study or handbook. This reference doesn't fit to your statement."

*Response:*

We thank the reviewer for the comment. We believe you mentioned to ref 37 in the text (Lars Dahlgren's book, now it's ref #40) but maybe you have mistakenly checked the reference list in the supplementary file.

21. "Table 1: Questions 1, 2 and 7 are very similar in content and objectives. Moreover, each of them focus on two or three issues at once. That can be limiting in gaining accurate answers from interviewees. Please consider that as a possible limitation in the construction of your interview guide together with the lacking deductive analytical framework. Dao-Tran et al 2018 doesn't provide that while Sakhon et al. 2017 is a framework for interventions. "

*Response:*

We thank the reviewer for the comment. Following Sekhon's framework, question 1 is designed to ask "Does the tool work?", question 2 asks "Do you like it?", and question 7 asks "Do you understand it?". We agree that these questions are very similar and could cause confusion for the respondents. However, during the interviews, MVQ explained each question in simple terms as stated above to guide the discussion effectively. Regarding your comments on the reference (similar to comment #8), we have clarified that we used Braun's references.

Editor's comments

- Along with your revised manuscript, please include a copy of the COREQ checklist for reporting of qualitative research, indicating the page/line numbers of your manuscript where the relevant information can be found (https://academic.oup.com/intqhc/article/19/6/349/1791966/Consolidated-criteria-for-reporting-qualitative)

*Response:*

We thank the reviewer for the comment. We provided the checklist.

- Please delete the section 'key messages'. Instead, please add a section entitled 'Strengths and limitations of this study', immediately after the abstract. This section should contain up to five short bullet points, no longer than one sentence each, that relate specifically to the methods. The novelty, aims, results or expected impact of the study should not be summarised here.

*Response:*

We thank the reviewer for the comment. We revised accordingly.

- Please provide more information on how participants were approached and recruited.

*Response:*

We thank the reviewer for the comment. We revised accordingly in the Informants section.

- Inspired by the work of the patient partnership strategy at The BMJ (https://www.bmj.com/campaign/patient-partnership), BMJ Open is encouraging active patient involvement in setting the research agenda. BMJ Open now requires authors of all submissions to the

journal to include a Patient and Public Involvement statement. The Patient and Public Involvement statement should be included as a sub-heading in the methods section of all manuscripts. It should provide a brief description of any patient involvement in study design or conduct of the study, as well as any plans to disseminate the results to study participants. If patients and or the public were not involved, please state "None". See our Instructions for Authors for further details: https://bmjopen.bmj.com/pages/authors/#reporting_patient_and_public_involvement_in_research.

*Response:*

We thank the reviewer for the comment. We added Patient and public involvement statement as None after the Acknowledgements in the text.

## VERSION 2 – REVIEW

| | |
|---|---|
| **REVIEWER** | Mazur, Ana<br>Bielefeld University, School of Public Health |
| **REVIEW RETURNED** | 26-Jun-2024 |

| | |
|---|---|
| **GENERAL COMMENTS** | Thank you for clarifying the open questions and considering my suggestions. |

## VERSION 2 – AUTHOR RESPONSE

**Reviewer: 1**

Dr. Ana Mazur, Bielefeld University

Comments to the Author:

Thank you for clarifying the open questions and considering my suggestions.

**Response:** Thank you.
